# Diagnosing Pheochromocytoma in the COVID-19 Era: A Case Report

**DOI:** 10.5811/cpcem.2022.2.55091

**Published:** 2022-07-26

**Authors:** Frank Mayer, Raafia Memon, Justin Stowens

**Affiliations:** * Christiana Care Health System, Department of Emergency Medicine, Newark, Delaware; † Christiana Care Health System, Department of Endocrinology, Newark, Delaware

**Keywords:** case report, pheochromocytoma, COVID-19, cardiomyopathy, paraganglioma

## Abstract

**Introduction:**

Pheochromocytomas and paragangliomas are rare neuroendocrine tumors that secrete catecholamines. Symptoms of these tumors are related directly to catecholamine excess but can be intermittent and easily misattributed to other, more common pathologies. Identification in the emergency department (ED) is inherently difficult. During the coronavirus 2019 (COVID-19) pandemic, physicians have had to account for both the disease itself as well as associated increased prevalence of cardiac, pulmonary, and vascular complications. Such shifting of disease prevalence arguably makes rarer diseases like pheochromocytoma less likely to be recognized.

**Case Report:**

We report a case of pheochromocytoma in a patient who presented to the ED in the fall of 2020, at a regional height of the COVID-19 pandemic, with complaints of fatigue, tachycardia, and diaphoresis. The differential diagnosis included pulmonary embolism, cardiomyopathy, congestive heart failure, and infectious causes. A broad workup was begun that included serology, electrocardiogram, computed tomography angiogram (CTA), and COVID-19 testing. Imaging was consistent with COVID-19 infection, and laboratory testing confirmed the diagnosis. A tiny retroperitoneal tumor was reported on CTA as “incidental” in the setting of multifocal pneumonia from severe acute respiratory syndrome coronavirus 2 infection. Additional history-taking revealed many years of intermittent symptoms suggesting that the tumor may have been more contributory to the patient’s presentation than originally suspected. Subsequent magnetic resonance imaging and surgical pathology confirmed the dual diagnosis of pheochromocytoma and COVID-19 pneumonia.

**Conclusion:**

This case presentation highlights the importance of careful history-taking, keeping a broad differential, and examining incidental findings in the context of the patient’s presentation.

## INTRODUCTION

Compared to other specialties, in emergency medicine patients are usually undifferentiated at the time of initial presentation. During the coronavirus 2019 (COVID-19) pandemic the overwhelming prevalence of patients presenting with COVID-19 has likely increased the occurrence of premature closure of the differential. This case presentation of a patient with a pheochromocytoma highlights this challenge and demonstrates that rare diseases should still be identified in the emergency department (ED). allowing patient care and referral to subspecialists to be expedited.

## CASE PRESENTATION

A 45-year-old woman with past medical history of anxiety, hypertension, and preeclampsia (10 years prior), presented to the ED with three weeks of transient fatigue and tachycardia that had worsened over the prior 24 hours. She additionally complained of intermittent tachypnea, dyspnea, chills, and profuse diaphoresis. The patient, who exercised regularly, was an active mother of three children. On the day before her ED presentation, she had run five miles on her treadmill without difficulty. Outpatient lab work from two weeks prior was remarkable for anemia, with hemoglobin of 7.0 grams per deciliter (g/dL) (reference range 11.7–15.7 g/dL). She had received an iron infusion the week prior to presentation, and symptoms had abated. However, when her dyspnea returned she presented to the ED wondering if a second iron infusion would help.

The patient was being evaluated during the regional peak of the COVID-19 pandemic prior to vaccine availability, and she had not been tested for COVID-19 previously. Notably, she reported abiding by social distancing guidelines and wearing a mask regularly.

**Image 1:**
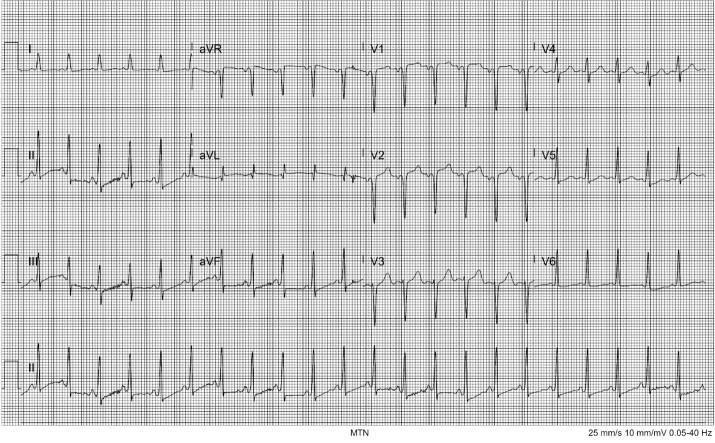
Electrocardiogram showing heart rate of 132 beats per minute, small ST-segment depressions in inferior leads with T-wave flattening in I and aVL.

Her triage vital signs were temperature of 36.8° Celsius, heart rate 137 beats per minute (bpm), respiratory rate 24 breaths per minute, blood pressure 173/132 millimeters of mercury (mm Hg), and peripheral oxygen saturation of 99% on room air. On physical exam she was anxious, speaking quickly and in earnest. She was notably diaphoretic. She was tachycardic but without murmurs, rubs, or gallops; 2+ pulses were palpated in all four extremities. Despite her dyspnea and tachypnea, her lungs were clear bilaterally with equal bilateral chest rise. She uncomfortably shifted her position frequently in the stretcher. An electrocardiogram (ECG) showed sinus tachycardia with ST-segment depression in the inferior leads with T-wave flattening in the lateral and inferior leads as well as a P-wave inversion and deep Q wave in V2 (Image 1).

**CPC-EM Capsule**

What do we already know about this clinical entity?

*Pheochromocytomas are rare and the prevalence of coronavirus disease 2019 (COVID-19) dwarfs them by comparison. Recognition is key as common medications used to control heart rate and blood pressure can worsen symptoms*.

What makes this presentation of disease reportable?

*The increased prevalence of cardiac, respiratory, and vascular complications associated with the COVID-19 virus make detection of rarer diseases more difficult during the pandemic*.

What is the major learning point?

*Keeping a broad differential can be difficult during COVID-19 surges. Avoid premature diagnostic closure and reexamine the differential as more data becomes available*.

How might this improve emergency medicine practice?

*This case reenforces the importance of a broad differential and demonstrates that making challenging diagnoses can and should be done in the emergency department*.

Preliminary workup was started for a differential diagnosis that included pulmonary embolism, acute coronary syndrome (ACS), anemia, and infectious etiologies including COVID-19. Intravenous fluids were given as well as 0.5 milligrams of IV lorazepam as the patient was experiencing severe anxiety. Labs were significant for only a mild anemia with a hemoglobin of 9.2 g/dL, and an elevated brain natriuretic peptide (BNP) of 3871 picograms per milliliter (pg/mL) (reference range 0–192 pg/mL). Troponin T was also noted to be < 0.01 nanogram per milliliter (ng/mL) (reference range 0.00–0.03 ng/mL). A two-view chest radiograph demonstrated mild multi-lobar infiltrates. Subsequent computed tomography angiogram (CTA) of the chest was negative for pulmonary embolism but did show evidence of pulmonary edema vs infection as well as a right-sided consolidation consistent with pneumonia. Multiple incidental findings including a small renal cyst, small liver cyst, and tiny mass in the left retroperitoneum were found on the CTA for which magnetic resonance imaging (MRI) and further follow-up was recommended. Testing for COVID-19 returned positive within a few hours. Clinician hand-off occurred at this point with a closed differential of severe COVID-19 infection.

**Image 2:**
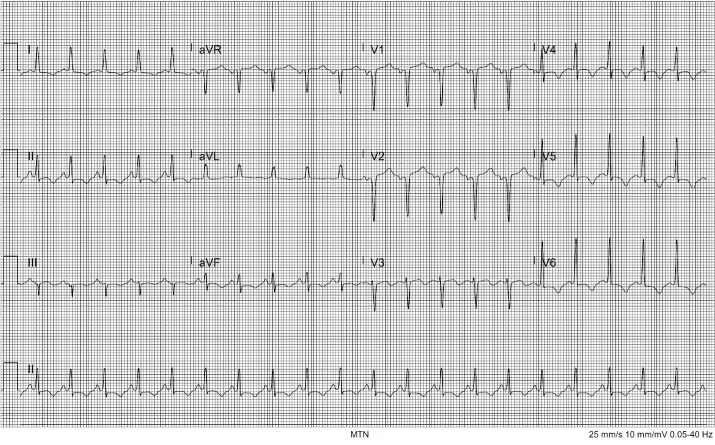
Electrocardiogram showing sinus tachycardia at 100 beats per minute; new T-wave inversions are noted in V4 though V6, as well as leads I, II, with T-wave flattening in aVF.

The patient improved greatly with fluids and benzodiazepines. Her heart rate decreased to just above 100 bpm, blood pressure decreased to 130/80 mm Hg, and all reported symptoms completely resolved at a two-hour reassessment. She was admitted to the hospital for further management. Pending transfer to the floor, an hour later she had a return of symptoms with palpitations and shortness of breath. Her vital signs were now notable for a return of tachycardia and tachypnea as well as elevated blood pressure. She remained afebrile. Repeat ECG showed new T-wave inversions in leads I, II, V4, V5, and V6 (Image 2).

Although not widely reported at this stage in the pandemic, concern for the possibility of a COVID-19-induced myocarditis was raised as well as new development of pulmonary embolism, pericardial effusion, or new occurrence of ACS. Elevated blood pressures were fortunately not consistent with dilated cardiomyopathy that has been seen with other viral pathogens. Initial troponin testing had also been negative. Serial troponin testing was sent, and a point-of-care cardiac ultrasound was performed. This demonstrated a hyperdynamic left ventricle with reduced ejection fraction of approximately 45%. Left ventricular wall thickness was increased and there were no signs of pericardial effusion, focal wall motion abnormality, or increased size of the right ventricle. Highly specific findings for right heart strain such as McConnell’s sign and decreased tricuspid annular plane systolic excursion were absent. As point-of-care cardiac ultrasound showed signs of heart failure without a dilated cardiomyopathy and serial troponin testing was negative, viral myocarditis did not seem likely.

During this re-evaluation further history was obtained. The patient revealed that she had been treated for hypertension intermittently over the prior nine years and that the pattern of sudden onset of symptoms with sudden resolution of symptoms had been occurring with less severity for many years. With the change in the patient’s clinical condition, an enhanced clinical history, dynamic vital sign changes, and bedside echocardio-graphy suggestive of heart failure, the clinical workup and differential was reviewed. The CTA showing the incidental retroperitoneal mass raised the possibility of a catecholamine-producing tumor, and this was discussed with the inpatient team. The patient was successfully stabilized with alpha blockade as well as beta blockade over the next few days.

Inpatient testing revealed elevated levels of plasma-free metanephrines, specifically norepinephrine at greater than 20 times the normal level, strongly suggestive of a neuroendocrine tumor. An MRI was performed demonstrating a 4.0 × 3.9 × 3.5-centimeter retroperitoneal mass consistent with pheochromocytoma (PCC). The patient was treated as well for COVID-19 pneumonia and medically optimized before discharge. As an outpatient she was evaluated by an endocrine surgeon and had subsequent adrenalectomy, after which pathology demonstrated a neuroendocrine tumor with capsular and vascular invasion. Following surgery, the patient had resolution of all symptoms and has not required further antihypertensive treatment.

## DISCUSSION

Paragangliomas and PCCs are part of the family of neuroendocrine tumors, with an exceedingly rare prevalence of between two and eight per million.^1^ Pheochromocytomas are catecholamine-producing tumors that arise from the adrenal medulla, while paragangliomas, their closely related counterparts, arise from non-head-and-neck sympathetic ganglia. They often produce more norepinephrine than epinephrine, but sometimes exclusively epinephrine. In contrast, paragangliomas produce mostly norepinephrine with some dopamine. Together they account for only 0.2–0.6% of all cases of severe hypertension in adults.^2^ Most tumors are benign, but those that are malignant are generally associated with familial genetic disorders.^3^ Malignancy is determined by metastases and local invasion.^1^ As in this case, up to 61% of tumors are found incidentally on imaging. 4–6

The classic triad of headache, diaphoresis, and tachycardia is associated with catecholamine tumors. However, these symptoms do not present together in a majority of patients. As seen in this case, the most common sign is paroxysmal or sustained hypertension, although 5–15% may be normotensive.^7^ Certain medications and physiologic stressors such as metoclopramide, anesthetics, and beta-blockers, trauma, surgery, or infection can precipitate symptoms. Cardiomyopathy is a rare complication and is attributed to the direct toxic effects of catecholamines on the myocardium and catecholamine-induced myocardial stunning, similar to the pathophysiology of takotsubo cardiomyopathy. It may be more common when PCC is untreated for long periods of time.^8^

Besides an example of rare pathology and an additionally rare occurrence of dual pathologies, this case offers an example of premature diagnostic closure. Premature diagnostic closure is a type of cognitive bias whereby the physician anchors on a diagnosis early in the clinical decision-making process and excludes other possible diagnoses, even when evidence supporting an alternative diagnosis is present. This can result in failure to make the correct diagnosis. It can also lead to diagnostic momentum where the incorrect diagnosis drives the ongoing workup and treatment plan further in the wrong direction, making correction more difficult.^9^ Keeping a broad early differential and reexamining that differential as new evidence becomes available helps avoid this type of error. Fortunately, in this case the early diagnostic closure was recognized when the patient’s clinical presentation worsened in a way not consistent with the working (incorrect) diagnosis of solitary COVID-19 infection.

This case specifically highlights an additional push toward early diagnostic closure due solely to the prevalence of COVID-19 disease during the local peak of the pandemic surge. For example, most patients with COVID-19 had a complaint of shortness of breath. It therefore became so likely that a complaint of shortness of breath was due to COVID-19 that it became difficult to consider attributing the shortness of breath to a different cause. In this case, the concomitant presence of COVID-19 made this push even stronger.

The novelty of the virus added yet another opportunity for early diagnostic closure. This patient presented during the stage of the pandemic when complications and varied presentations of the infection were still being discovered. Complications such as the relationship between the infection and thromboembolic disease were yet to be confirmed.^10^ In such a setting, unique or rarer complaints of patients with COVID-19 might be inappropriately attributed to the infection as simply “under-reported” when in truth they were due to a different disease process. In our patient, the transient nature of the patient’s hypertension and dyspnea were initially incorrectly attributed to COVID-19.

The presence of the COVID-19 infection resulted in premature diagnostic closure. It should be noted that the difficulties with making this diagnosis extended beyond the cognitive challenge. With MRI resources kept out of routine use due to extensive cleaning needed between COVID-19 patients, there was significant delay in completing the MRI. In fact, the patient’s urinary metanephrine test (a 10-day, send-out test) resulted on the same day that the patient finally received the MRI.

## CONCLUSION

Pheochromocytomas are rare and have signs and symptoms that typically arise from more common causes, making them a difficult diagnosis in the ED in non-pandemic times. As always in emergency medicine, careful history-taking, considering the patient’s entire constellation of symptoms, and keeping a broad differential is critical to making the diagnosis. In the setting of COVID-19 these tasks become equally more paramount and more difficult. Cases such as the one we report here highlight the need for heightened suspicion and diligence during this time of diagnostic obfuscation caused by the COVID-19 pandemic.
